# Reply to Kudus, A.L. Comment on “Brandl et al. Immediate Effects of Myofascial Release on the Thoracolumbar Fascia and Osteopathic Treatment for Acute Low Back Pain on Spine Shape Parameters: A Randomized, Placebo-Controlled Trial. *Life* 2021, *11*, 845”

**DOI:** 10.3390/life12060868

**Published:** 2022-06-10

**Authors:** Andreas Brandl, Christoph Egner, Robert Schleip

**Affiliations:** 1 DIPLOMA Hochschule, 37242 Bad Sooden-Allendorf, Germany; brandl.andreas@stud.diploma.de (A.B.); christoph.egner@diploma.de (C.E.); 2 Conservative and Rehabilitative Orthopedics, Department of Sport and Health Sciences, Technical University of Munich, 80333 Munich, Germany

One comment [1] was submitted on our published article [2]. The author pointed out valuable reservations on some method-specific points, especially on the accuracy of the video raster stereography system (VRS; DIERS Formetric 4D, DIERS International GmbH, Germany). We are pleased to respond to the author’s astute objections and the opportunity to specify our methodological considerations.

First, we would like to note some quotes in the commentary that were not accurately or incorrectly reproduced. The author claimed that we were hypothesizing that myofascial release techniques (MFR) have an effect on spinal shape in chronic low back pain. This was not interpreted correctly by the commentator. Our work examined the effects of MFR (and osteopathic treatment) in patients with acute low back pain (aLBP). Furthermore, the author copied a table and misrepresented the contents. The value for the outcome “Kyphotic angle (degrees)” in the MFR group was −8.32, and not 8.32 as quoted.

There was concern about the clinical implication of our results in relation to pain not measured after intervention using the visual analog scale (VAS). This was outside the scope of our work, and we agree that we cannot provide information on longer-term changes in spinal shape parameters, which should be the task of future work. Measurement of pain with the VAS and the Oswestry Disability Questionnaire in the German version was part of our enrollment procedure to verify the inclusion criteria of aLBP as defined by the European Guidelines for the Treatment of aLBP [3]. We emphasized this point in our title “Immediate Effects (…)” as well as in our limitations section in the discussion.

The author questioned “how meaningful is the VRS significant kyphotic difference of 8 degrees in this study?”, following the work of Bassani et al. [4]. The mentioned VRS uncertainty of 18 degrees indicates the difference between the Cobb angle determined by the software for semi-automatic evaluation of radiological images (RAD; sterEOS software, EOS Imaging, France) and the VRS at baseline. This parameter was not used in our study and is, therefore, not applicable. Instead, we examined the functional leg length discrepancy, kyphosis angle and lordosis angle. A close reading of this paper shows a strong correlation (r = 0.7) between the kyphosis angle determined by RAD and the VRS with only 1 degree difference in measurement. However, the work of Bassani et al. has some limitations that make it inapplicable to our randomized placebo-controlled trial:

- They studied only adolescent subjects (mean age 13 ± 2 years) with and without scoliotic curvatures (80% versus 20%). The sample included 24% with severe scoliosis and a Cobb angle greater than 40°. This makes the sample inhomogeneous and not comparable to our study population with a mean age of 43.8 ± 10.5 years and aLBP.
- Bassani et al. examined their subjects during an orthopedic visit over three years and calculated the Pearson or Spearman correlation (r) in 192 participants. In this context, r is not a reliable indication of causality, as other variables may influence the correlation [5]. Therefore, Koo et al. [6] strongly recommend the use of the intraclass correlation coefficient (ICC) and point out that r is not an ideal measure of reliability.
- Although the authors included 192 participants, they only used a small group of 30 subjects for follow-up. For this group, they calculated the measurement difference for the Cobb angle between baseline and 6 months later to infer the within-subjects correlation [7]. Both RAD and VRS showed equal mean differences (0° ± 7° versus 1° ± 9°) and were not statistically significantly different from zero. The correlation plot shows a wide scatter of data, with 16 of 30 data points falling outside the 95% confidence interval. The Bland–Altman method of calculating correlation coefficients for repeated observations is based on the General Linear Model and the Analysis of Covariance. Standard assumptions for using these statistical methods are normally distributed data, homogeneity of variance and a relationship between the dependent variable and the covariate. The model is also very sensitive to outliers. Bassani et al. did not specify any of these assumptions. From the graphs presented, it appears that there is an outlier and the data is apparently not normally distributed, which implies a high risk of bias in the calculation.

Because of the limitations of the Bassani et al. paper mentioned above, we chose to use the reliability data from Degenhardt et al. [8], which provided not only ICC for our measured parameters, but even values for the smallest detectable change. We agree that validity and reliability of the measurement system is essential to the meaningfulness of a study. In this way, we evaluated the work of Degenhardt et al. using the Guidelines for Reporting Reliability and Agreement Studies (GRRAS) and followed the recommendations of Gerke et al. [9]. We found agreement for all 15 items of the GRRAS checklist, indicating the high quality of this reliability study. In 2020, Krott et al. [10] presented a comprehensive meta-analysis of the validity and reliability of raster stereographic measurements of spinal posture. They found reliability overall effect sizes of rs > 0.91, ps < 0.001, and validity overall effect sizes of rs > 70, ps < 0.001, compared with traditional radiological imaging. Therefore, we consider our results based on VRS to be valid and reliable as well.

In his commentary, the author urged, “we need more qualitative research to build a good scientific the foundation for MFR treatments”. Many studies have now been published on the effectiveness of MFR. The National Library of Medicine lists four meta-analyses on this topic for the past two years alone. Chen et al. [11] confirm that MFR has a significant effect on reducing low back pain in patients with low back pain. Ughreja et al. [12] found improvements in pain, sleep and quality of life in patients with fibromyalgia. However, the effects on biomechanical parameters found by Burk et al. [13] and the mechanisms behind them are poorly understood, which is why we conducted our study. Spinal shape parameters changed significantly in our treatment groups. These values exceeded even the smallest detectable changes in more than three quarters of the cases.

In conclusion, we fully agree with the author that further research is needed to also investigate the implications of our results, e.g., proprioceptive, neurovascular and neuromuscular aspects. We are, therefore, very grateful for the valuable comment and hope that we have been able to elaborate on our methodological considerations that underpin the validity and reliability of the VRS, as well as our thoroughly investigated results.

## Data Availability

Data can be made available by the author upon request.
